# On the relationship between root economics and plant hydraulic traits

**DOI:** 10.1111/nph.71442

**Published:** 2026-07-13

**Authors:** Anvar Sanaei, Srijna Saxena, Kevin E. Mueller, M. Luke McCormack, Bernhard Schuldt, Daniel C. Laughlin, Bruno H. P. Rosado, Grégoire T. Freschet, Shalom D. Addo‐Danso, Kathryn E. Barry, Joana Bergmann, Nico Eisenhauer, Florentin C. Jaeger, Hendrik Poorter, Harry Olde Venterink, Jorad De Vries, Monique Weemstra, Liesje Mommer, Alexandra Weigelt

**Affiliations:** ^1^ Institute of Biology Leipzig University Leipzig 04103 Germany; ^2^ German Centre for Integrative Biodiversity Research (iDiv) Halle‐Jena‐Leipzig Leipzig 04103 Germany; ^3^ Department of Biological, Geological, and Environmental Sciences Cleveland State University 2121 Euclid Avenue Cleveland 44115 USA; ^4^ Center for Tree Science The Morton Arboretum 4100 Illinois Route 53 Lisle IL 60532 USA; ^5^ Forest Botany TUD Dresden University of Technology Pienner Straße 7 Tharandt 01737 Germany; ^6^ Department of Botany University of Wyoming 1000 E. University Ave Laramie WY 82071 USA; ^7^ Department of Ecology, IBRAG Universidade do Estado do Rio de Janeiro (UERJ), R. São Francisco Xavier 524, PHLC, Sala 220, Maracanã Rio de Janeiro 20550900 RJ Brazil; ^8^ Theoretical and Experimental Ecology Station, CNRS, 2 route du CNRS Moulis 09200 France; ^9^ CSIR‐Forestry Research Institute of Ghana PO Box UP 63 KNUST Kumasi Ghana; ^10^ Ecology and Biodiversity, Department of Biology Utrecht University Padualaan 8 Utrecht 3584 CH the Netherlands; ^11^ Leibniz Centre for Agricultural Landscape Research (ZALF) Eberswalder Straße 84 Müncheberg 15374 Germany; ^12^ School of Integrative Plant Science Cornell University 236 Tower Road Ithaca NY 14850 USA; ^13^ Horticulture and Product Physiology Wageningen University and Research AA Wageningen 6700 the Netherlands; ^14^ Vrije Universiteit Brussel Research Group WILD Pleinlaan 2 Brussels 1050 Belgium; ^15^ Forest Ecology and Forest Management Group Wageningen University & Research PO Box 47 Wageningen 6700 AA the Netherlands

**Keywords:** drought resistance, plant hydraulic traits, plant nutrient strategies, root collaboration gradient, root conservation gradient, root economics space, safety‐efficiency trade‐off, woody species

## Abstract

- Drought stress constrains the productivity of terrestrial ecosystems and the distribution of plant species. To withstand drought stress, plants have evolved diverse water‐use strategies. Despite the critical function of roots in whole‐plant water regulation, drought strategies have been primarily studied from an aboveground perspective, and so our knowledge of how fine‐root traits are related to aboveground hydraulic traits remains limited.
- Here, we compiled a dataset of primarily woody species comprising five aboveground plant hydraulic traits associated with water‐use strategies and four fine‐root traits from the root economics space (RES). We investigated how the global diversity in ecological strategies of the RES traits relates to aboveground hydraulic traits contributing to drought resistance.
- We found a slight trend towards acquisitive species with higher root nitrogen content and lower root tissue density having lower drought resistance in aboveground tissues. Species with thicker roots displayed a tendency towards slightly higher drought resistance than thin‐rooted species.
- The weakness of these relationships suggests that aboveground drought adaptations are largely independent of classical axes of fine root ecological strategies, pointing to either a decoupling between aboveground and belowground responses or a stronger coordinating role for other root traits such as maximum rooting depth or root cortex fraction in hydraulic adaptation.

## Graphical abstract

Conceptual summary figure showing the results of the relationships between aboveground plant hydraulic strategies and root economics space (RES) traits.

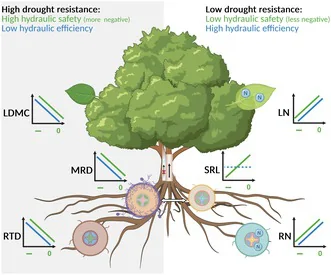

## Introduction

Water is essential for the survival of all life forms, and drought is a major cause of plant stress that limits ecosystem productivity and plant species distribution (Müller & Bahn, 2022; Torres‐Ruiz *et al*., 2024). As climate change progresses, the impact of drought on plants is increasing at unprecedented rates (Chen *et al*., 2025). Plants have evolved a wide range of strategies to cope with drought, such as drought resistance or dehydration tolerance (Volaire, 2018; Bristiel *et al*., 2019), to avoid hydraulic failure and carbon starvation (Choat *et al*., 2018). A growing body of studies has linked these strategies to aboveground hydraulic traits of leaves and stems in woody species (Kröber *et al*., 2014; Blackman *et al*., 2019; Rosas *et al*., 2019). Despite the tight coordination of aboveground hydraulic traits with stomatal regulation (McCulloh *et al*., 2019), water uptake fundamentally depends on roots and their access to soil water (Cuneo *et al*., 2016; Lozano *et al*., 2020). However, in addition to root system architecture and extent (Tumber‐Dávila *et al*., 2022; Laughlin *et al*., 2023), the role of fine‐root traits in drought strategies remains uncertain (Comas *et al*., 2013), likely due to the inherent difficulty of monitoring belowground processes. Moreover, there has been a long‐standing assumption that stomata are the principal determinants of plant responses to water stress (Martin‐StPaul *et al*., 2017). Here, we study how fine‐root traits of primarily woody species are linked to selected aboveground hydraulic traits to gain a whole‐plant perspective on drought strategies of plants.

Plant hydraulic traits are defined as traits involved in plant water status, transport and storage (Griffin‐Nolan *et al*., 2018). Some key hydraulic traits that play an important role in drought resistance based on recent literature (Bartlett *et al*., 2012; Anderegg, 2015; D. Liu *et al*., 2024) and expert knowledge include the xylem water potential at 50% loss of hydraulic conductivity (P_50_), leaf water potential at turgor loss point (π_tlp_), maximum xylem conductivity per unit sapwood area (K_s_), the leaf‐to‐sapwood area ratio (A_l_ : A_s_), and wood density (WD) (Table 1). Traits such as P_50_ and π_tlp_ are classified as hydraulic safety traits (Fuchs *et al*., 2021) because they reflect a plant's resistance to embolism (Gleason *et al*., 2016) and its capacity to maintain water transport under stress. Plants with more negative P_50_ and π_tlp_ are associated with higher drought resistance (Bartlett *et al*., 2012; Anderegg *et al*., 2016). By contrast, K_s_ and A_l_ : A_s_ represent hydraulic efficiency traits (Gleason *et al*., 2016; Mencuccini *et al*., 2019), as they quantify a plant's ability to transport water through the xylem (Li *et al*., 2023). Plants with higher K_s_ and A_l_ : A_s_ are associated with lower drought resistance (Hoeber *et al*., 2014; Mencuccini *et al*., 2019). Studies on hydraulic traits have a strong focus on traits of woody species, including wood density (WD), which is used as a predictor of capacitance (Ziemińska *et al*., 2020), reflecting elastic storage of water in living cells (Cruiziat *et al*., 2002). Plants with higher WD are associated with higher drought resistance (Greenwood *et al*., 2017).

**Table 1:** Definition of plant functional traits considered in this study.

Traits	Abbreviation	Definition	Unit
Aboveground plant hydraulic traits			
Xylem water potential at 50% loss of conductivity	P_50_	Xylem water potential at which 50% of hydraulic conductivity is lost	MPa
Leaf water potential at turgor loss point	π_tlp_	Leaf water potential at which average cell turgor is lost and leaf wilting occurs	MPa
Maximum stem xylem‐specific hydraulic conductivity	K_s_	Rate of sap‐flow per cross‐sectional area of the xylem across a given pressure gradient	kg m^−1^ MPa^−1^ s^−1^
Leaf area to sapwood area ratio	A_l_ : A_s_	Ratio of summed leaf area to conductive xylem area of a branch	cm^2^ cm^−2^
Inverse of wood density	Inverse WD	Inverse wood density is defined as the green volume divided by oven‐dry mass	cm^3^ g^−1^
Root economics space traits			
Root nitrogen content	RN	Mass of nitrogen per root dry mass	mg g^−1^
Root tissue density	RTD	Dry mass of fine root per unit volume of fresh root	g cm^−3^
Mean root diameter	MRD	Average of all root diameter observations of a root diameter distribution	mm
Specific root length	SRL	Length of a root per unit dry mass	m g^−1^

Recent work has highlighted the existence of a two‐dimensional root economics space (RES) across species (Bergmann *et al*., 2020; Matthus *et al*., 2025), in which variation in root traits partly aligns with aboveground economics traits (Weigelt *et al*., 2021). The first gradient of the RES, the conservation gradient, separates acquisitive species with inexpensive and more metabolically active tissue and high root nitrogen (RN) content from conservative species that invest in high root tissue density (RTD, root mass per root volume). This gradient is conceptually similar to the leaf economics spectrum (LES), which ranges from inexpensively constructed leaves optimized for fast resource acquisition to persistent leaves with a slow rate of carbon return (Wright *et al*., 2004; Reich, 2014). The second gradient of the RES, the collaboration gradient, ranges from ‘do‐it‐yourself’ soil exploration by thin roots with high specific root length (SRL, root length per root mass) to ‘outsourcing’ by investing carbon into roots with higher mean diameter (MRD), offering a larger cortex fraction to the mycorrhizal partners, which efficiently forage for resources (Bergmann *et al*., 2020). Both gradients – conservation and collaboration – are intrinsically linked to both nutrient and water uptake and carbon allocation in plants, and thus their key traits could be related to plant strategy towards drought.

There is evidence suggesting that fine‐root economics traits may play a crucial role in alleviating drought stress (Comas *et al*., 2013). For example, higher SRL was associated with enhanced specific hydraulic conductance in Mediterranean herbaceous species (Hernández *et al*., 2010) and enabled temperate trees to maintain higher rates of sap flow during summer drought (Tran *et al*., 2026). Similarly, a study on temperate grassland species showed that higher RTD confers greater drought resistance (Garbowski *et al*., 2025). Additionally, for some conifer species, it has been shown that with higher MRD and RN, root hydraulic conductivity decreases, while it increases with higher RTD (Masumoto *et al*., 2022). Furthermore, MRD is positively associated with arbuscular mycorrhizal colonization, which generally promotes water uptake under dry conditions and improves drought resistance (Moora *et al*., 2025).

**Fig. 1:**
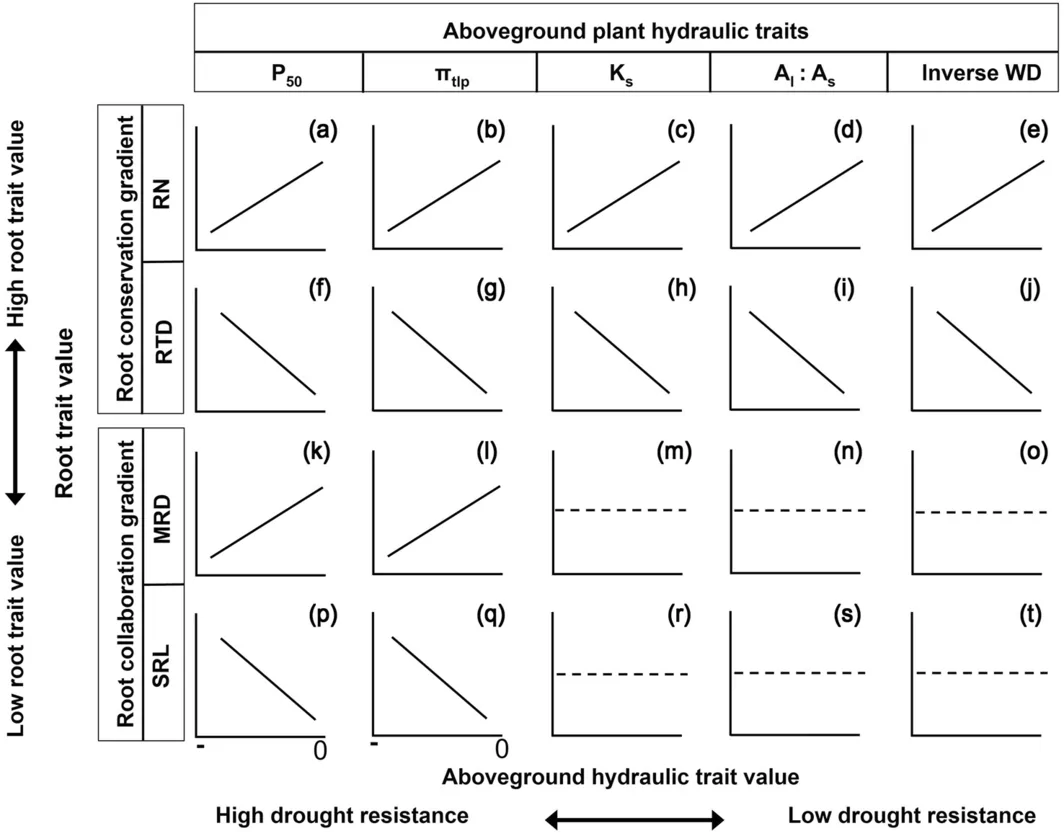
Conceptual figure outlining the expected associations and hypotheses related to aboveground plant hydraulic traits and the root economics space traits. Each panel shows the predicted relationship between each pair of traits. Solid lines denote significant predicted relationships, and dashed straight lines denote nonsignificant predicted relationships between each pair of traits. Note that only linear relationships (regardless of differences in slope) are considered. Columns 1–4 show the relationships of P_50_, π_tlp_, K_s_, A_l_ : A_s_ and inverse WD, respectively, with RN (a–e), RTD (f–j), MRD (k–o) and SRL (p–t). High drought resistance is defined by more negative hydraulic safety traits (P_50_ and π_tlp_) values as well as lower hydraulic efficiency traits (K_s_ and A_l_ : A_s_), whereas low drought resistance is defined by less negative hydraulic safety traits and higher hydraulic efficiency traits values. π_tlp_, leaf water potential at turgor loss point; P_50_, xylem vulnerability to embolism; π_tlp_, leaf water potential at turgor loss point; K_s_, maximum stem xylem‐specific hydraulic conductivity; A_l_ : A_s_, leaf area to sapwood area ratio; inverse WD, inverse wood density; RN, root nitrogen content; RTD, root tissue density; MRD, mean root diameter; and SRL, specific root length. Note that P_50_ and π_tlp_ values are negative.

In this study, we hypothesize pairwise relationships between the four RES traits and the five key aboveground hydraulic traits mentioned above based on existing evidence and first principles as detailed below (Fig. 1). We focus primarily on woody species, where data availability is substantially greater. While the potential importance of coordination between root economics traits and hydraulic traits has been highlighted, it is still unclear whether and how strongly these traits are correlated across species. Some relationships may be direct, for example, resulting from the scaling of xylem conduit dimensions in roots with root diameter (Kirfel *et al*., 2017). However, most links between aboveground hydraulics and RES traits are likely indirect and thus potentially weak. For instance, following whole‐plant trait economics (Reich, 2014), species with conservative root economic traits classified as ‘slow’ (e.g. high RTD and low RN) may be more likely to have conservative aboveground hydraulic traits (e.g. low K_s_ and A_l_ : A_s_, more negative P_50_ and π_tlp_) in order to synchronize the pace of aboveground and belowground resource uptake and transport capacities, while maximizing organ lifespan. By contrast, the collaboration gradient optimizes resource uptake on both ends using differing strategies (i.e. by either very fine roots or their fungal partners), so a trait like SRL might be less tightly related to aboveground hydraulic traits.

### Root nitrogen (RN) content – ‘acquisitive’

RN is supposed to be functionally equivalent to leaf nitrogen (LN) with regards to growth rate and metabolic activity (Weigelt *et al*., 2021). Higher LN permits higher maximum rates of photosynthesis, which in turn requires higher maximum rates of stomatal conductance (Xiong & Flexas, 2020). It has also been shown that hydraulic efficiency traits (K_s_ and A_l_ : A_s_) are positively associated with LN (D. Liu *et al*., 2024). Therefore, we assume the same positive relationships of hydraulic efficiency traits with RN (Fig. 1c,d). Furthermore, plants cannot simultaneously optimize both hydraulic efficiency and hydraulic safety traits; that is, there is a weak efficiency‐safety trade‐off (Gleason *et al*., 2016; Fuchs *et al*., 2021). Based on this trade‐off, species with higher hydraulic efficiency are associated with higher LN and RN and should tend to have lower hydraulic safety (less negative P_50_ and π_tlp_; Fig. 1a,b). This would corroborate earlier findings where plants with higher RN have shown lower drought resistance (Salpagarova *et al*., 2014; de Vries *et al*., 2016).

### Root tissue density (RTD) – ‘conservative’

Unlike the acquisitive resource strategy, species with a conservative resource strategy are thought to have a higher water use efficiency, for example, through water‐saving behaviour (Fichot *et al*., 2009). From an aboveground perspective, it is known that species with more conservative economic traits (e.g. higher leaf dry matter content (LDMC) or WD) often exhibit xylem that is more resistant to embolism and has lower hydraulic efficiency (McCulloh *et al*., 2019; Májeková *et al*., 2021). Additionally, LDMC is also associated with more negative π_tlp_ (Blumenthal *et al*., 2020). Belowground, species with higher RTD tend to have smaller xylem conduits (Yamauchi *et al*., 2021), which can result in a reduced hydraulic conductance in both roots and stems and, ultimately, a lower risk of embolism formation (Fu & Meinzer, 2019; Isasa *et al*., 2023). As a result, higher RTD supports greater drought resistance (Garbowski *et al*., 2025). Based on these patterns, we expected that RTD should be associated with greater resistance to embolism (more negative P_50_ and π_tlp_) (Fig. 1f,g) and higher WD (Fig. 1j) while having a lower hydraulic efficiency (Fig. 1h,i).

### Mean root diameter (MRD) – ‘outsourcing’

The relationship between MRD and drought resistance is complex and can vary in direction depending on the underlying mechanisms (Kou *et al*., 2022). Generally, thick‐rooted species tend to have larger conduits (Kong *et al*., 2019), resulting in more efficient water conductance and hence increased xylem conductivity (Tyree & Zimmermann, 2013). However, larger conduits have been shown to be more susceptible to embolism (Domec *et al*., 2010; Isasa *et al*., 2023), thus potentially increasing the risk of embolism formation leading to lower safety (but see Lübbe *et al*., 2022). As MRD increases, the cortex‐to‐stele ratio also increases, leading to proportionally more cortex in thicker roots (Kong *et al*., 2019), therefore higher mycorrhizal colonization and hence higher water uptake capacity under dry conditions (Moora *et al*., 2025). Thicker roots can also improve soil penetration in compact soils (Lynch *et al*., 2021), which could be advantageous for water uptake during drying. By contrast, thicker roots may also be more prone to decreasing plant–soil connectivity during dry conditions and thus losing water uptake capacity because their larger cortex area may result in greater water loss and root shrinkage away from the soil surface (Schuldt *et al*., 2025). Thus, higher root shrinkage might effectively reduce water uptake but also stop water loss to dry soil without the need to adjust hydraulic conductance. We therefore hypothesize that species with higher MRD tend to have lower hydraulic safety (less negative P_50_ and π_tlp_; Fig. 1k,l). However, due to the contrasting effect of MRD on hydraulic conductivity and a lack of studies linking MRD to WD, we have no predictions for hydraulic efficiency and WD (Fig. 1m–o).

### Specific root length (SRL) – ‘do‐it‐yourself’

Similar to MRD, the relationship between SRL and drought resistance is equally variable, with studies showing higher (Comas *et al*., 2013; Tran *et al*., 2026) or lower drought resistance (Garbowski *et al*., 2025) potentially due to different mechanisms. Species with high SRL tend to have thinner roots with smaller conduits. These species are associated with lower risk of embolism formation, lower hydraulic conductance and lower cortex‐to‐stele ratios. These may lead to lower root shrinkage and consistently higher plant–soil contact during dry conditions. The latter could facilitate continued access to residual water under dry conditions, which would require lower (more negative) P_50_ and π_tlp_ to enable water uptake from dry soil. In addition, thinner roots are reported to have thicker cell walls, which could lead to higher xylem safety (Pratt *et al*., 2007). Independent of the MRD itself, higher SRL leads to a higher root surface area to soil volume ratio at constant root mass (Freschet *et al*., 2021), which enhances root‐soil contact at the whole plant level, thereby improving water uptake (Comas *et al*., 2013). All these mechanisms would indicate an increased drought resistance with higher SRL. However, recent studies suggest that species with higher root surface area (high SRL) can lead to higher K_s_ (Cai *et al*., 2022) or lower drought resistance (Garbowski *et al*., 2025), which contradicts these expectations. Due to these mechanisms operating in contrasting directions, we anticipate only weak relationships between SRL and hydraulic efficiency traits (Fig. 1r,s) while we hypothesize a negative relationship between SRL and hydraulic safety traits (Fig. 1p,q). Due to the lack of literature, we do not expect direct relationships between SRL and WD (Fig. 1t).

To summarize, our study integrates traits from the RES with key aboveground hydraulic traits to investigate whole‐plant drought alleviation strategies. The main aim of this study is to use aboveground hydraulic traits as an interface to link individual RES traits, rather than the two‐dimensional trade‐offs themselves. We hypothesised that species with higher RN (the fast end of the conservation gradient) would be associated with higher hydraulic efficiency (K_s_ and A_l_ : A_s_) and lower hydraulic safety (P_50_ and π_tlp_) traits. By contrast, we hypothesised that species with higher RTD (the slow end of the conservation gradient) would be associated with lower hydraulic efficiency but greater hydraulic safety. We also hypothesised that species with higher MRD (the outsourcing end of the collaboration gradient) tend to have lower hydraulic safety traits, whereas species with higher SRL (the do‐it‐yourself end of the collaboration gradient) would follow the opposite pattern and be associated with greater hydraulic safety (see Fig. 1 for a depiction of our hypotheses).

## Materials and Methods

### Data compilation

#### Plant hydraulic traits

We searched the literature for data on aboveground plant hydraulic traits, primarily those associated with drought resistance. These traits included stem xylem vulnerability to embolism (P_50_, MPa), leaf water potential at turgor loss point (π_tlp_, MPa), sapwood area‐specific hydraulic conductivity (K_s_, kg m^−1^ MPa^−1^ s^−1^), leaf‐to‐sapwood area ratio (A_l_ : A_s_) and wood density (WD, g cm^−3^). To survey the literature, we used ‘xylem vulnerability to embolism’, ‘xylem cavitation’, ‘leaf water potential at turgor loss point’, ‘leaf water potential’, ‘xylem hydraulic conductivity’, ‘Huber value’, ‘leaf area to sapwood area ratio’, ‘wood density’ and ‘stem‐specific density’ as keywords. We conducted our search in Web of Science, which includes ISI Web of Knowledge (http://webofknowledge.com; cut‐off date 30 October 2024) and Google Scholar (http://scholar.google.com; cut‐off date 30 October 2024), as well as the available xylem functional traits database (Choat *et al*., 2012) (https://xylemfunctionaltraits.org; cutoff date 30 October 2024) and the TRY Plant Trait Database (Kattge *et al*., 2020). More specifically, we supplemented the data for wood density and/or stem‐specific density (stem dry mass per stem fresh volume) using the TRY dataset when data were unavailable from the original compiled sources. Hence, most of our data were acquired from previously published scientific papers – including meta‐analyses, syntheses and research data papers (Blackman *et al*., 2010; Kröber *et al*., 2014; Anderegg, 2015; Yin *et al*., 2018; Zhu *et al*., 2018; Barros *et al*., 2019; Liu *et al*., 2019; He *et al*., 2020; Laughlin *et al*., 2020; Sanchez‐Martinez *et al*., 2020; Tan *et al*., 2020; Anderegg *et al*., 2021; Májeková *et al*., 2021; Matos *et al*., 2021; Medina‐Vega *et al*., 2021; Rosas *et al*., 2021; Vargas *et al*., 2021, 2022; Xu *et al*., 2021; Guillemot *et al*., 2022; Serra‐Maluquer *et al*., 2022; Yi *et al*., 2022; Isasa *et al*., 2023; Jin *et al*., 2023; Tavares *et al*., 2023; Zhu & Zhao, 2023; Ziegler *et al*., 2023; Augustine & McCulloh, 2024; D.‐L. Huang *et al*., 2024; Decarsin *et al*., 2024; Hu *et al*., 2024; R. Huang *et al*., 2024; X. Liu *et al*., 2024; H. Liu *et al*., 2024; Sterck *et al*., 2024) – in which most of the meta‐analyses and syntheses used the TRY Plant Traits Database (Kattge *et al*., 2020). To avoid using data with methodological artefacts when measuring stem vulnerability to embolism (Schuldt *et al*., 2025), we only included sigmoidal vulnerability curves and excluded the air‐injection double‐end and single‐end measurement methods for P_50_ (Cochard *et al*., 2013), and across all species, we filtered out P_50_‐values that were less negative than −1.0 MPa. In contrast to (Guillemot *et al*., 2022) that applied a threshold of −0.5 MPa as they covered tropical species only, we decided to filter out all values less negative than −1.0 MPa as reported P_50_ values above this threshold commonly were produced by methods prone to the open‐vessel artefact (Choat *et al*., 2010; Cochard *et al*., 2010; Martin‐StPaul *et al*., 2014; Jansen *et al*., 2015; Schuldt *et al*., 2025). Accordingly, when both π_tlp_ and P_50_ of the same species were present in our database, then π_tlp_ with more negative values than P_50_ were filtered out (Sergent *et al*., 2020). For multiple observations of the same species, we used the average value per trait per species. We used the inverse WD as a proxy for capacitance (Ziemińska *et al*., 2020) throughout all the analyses. As a result, the compiled data mainly covers woody plants (*n* = 508); however, there were some nonwoody (*n* = 116) data included for π_tlp_ (*n* = 111) and inverse stem‐specific density (inverse SSD, *n* = 44).

#### Economic traits of roots and leaves

We focused on four core traits used within the RES (Bergmann *et al*., 2020): RN content (mg g^−1^), RTD (g cm^−3^), mean root diameter (MRD, mm) and SRL (m g^−1^). For root traits, we used the Global Root Trait (GRooT) database (Guerrero‐Ramírez *et al*., 2021), which provides species mean trait values. The GRooT database gathered the trait data from species pools of fine‐root traits, < 2 mm in diameter, that differ in root diameter cut‐off (root order); thus, species that are included in this study might not have the same diameter cut‐off, for example, root orders. We also included additional root data from the literature for those species where we had available compiled aboveground plant hydraulic trait data but where root data were missing (Vleminckx *et al*., 2021; Yan *et al*., 2022; Liang *et al*., 2023; Guilbeault‐Mayers & Laliberté, 2024; Jimoh *et al*., 2024; Langguth *et al*., 2024; Wang *et al*., 2024; Zheng *et al*., 2024; Sanaei *et al*., 2026). Furthermore, we acquired data on two main LES traits, LN and LDMC, from the TRY Plant Trait Database (Kattge *et al*., 2020) to examine how functionally equivalent leaf and root traits (i.e. LN and RN; LDMC and RTD) relate to plant hydraulic traits.

### Data processing

All the analyses were performed using the R software (R Core Team, 2024). We first matched the species name from the accepted nomenclature of the World Checklist of Vascular Plants (WCVP) using the *wcvp_match_names* function of the rWCVP package (Brown *et al*., 2023). We then classified each species as woody or nonwoody, using the more recent woodiness database (Poppenwimer *et al*., 2023). We also assigned a climate zone to each species based on the WCVP database (Govaerts *et al*., 2021). For those species without climate information in the WCVP database, supplementary data from the Plants of the World Online website were used.

### Statistical analyses

To meet the normality assumptions, all traits were log‐transformed before the analyses. To do so, we used log() for K_s_, A_l_ : A_s_ and WD as well as the RES traits because the original values only contained positive values. However, to handle the negative values of P_50_ and π_tlp_, we used the Yeo–Johnson transformation (Yeo & Johnson, 2000) using the *step_YeoJohnson* function of the recipes package (Kuhn *et al*., 2026). To explore the trait associations, we assessed univariate relationships between the hydraulic traits and the RES traits using ordinary least squares (OLS) regression. However, to account for potential evolutionary dependence on univariate relationships between the plant hydraulic traits and the RES traits, we also performed phylogenetic generalized least squares (PGLS) regression using the function of *pgls* in the caper package with lambda estimated with maximum likelihood (Orme *et al*., 2023). To do so, the phylogenetic tree for all species was first constructed using the *phylo.maker* function in the V.PhyloMaker package (Jin & Qian, 2019). Further, pairwise correlations among all the RES and hydraulic traits were tested using Pearson correlations and visualised using the *ggraph* function of the ggraph package (Pedersen, 2022). The pairwise correlations allow us to assess not only the correlation between the RES and hydraulic traits but also the magnitude, strength and direction of correlations within both the hydraulic traits and the RES traits. To complement the relationships between hydraulic traits and the RES traits, we tested whether the relationships between hydraulic traits and both LN and LDMC align with those observed for RES traits, particularly RN and RTD. While exploring the data, we found a clear separation between woody and nonwoody species in both the RES and the two aboveground plant hydraulic traits (leaf π_tlp_ and inverse WD); thus, we performed separate analyses for woody and nonwoody species. The relationships between aboveground hydraulic traits and the RES traits for woody species are reported in the main file, while the bivariate regression analyses between the two hydraulic traits (π_tlp_ and inverse SSD) and the RES traits for nonwoody species are shown in the supplementary information.

## Results

Overall, the final aggregated data on species‐level plant hydraulics, comprising measurements for at least one of the RES traits, consisted of P_50_ (*n* = 363), π_tlp_ (*n* = 248), K_s_ (*n* = 325), A_l_ : A_s_ (*n* = 272) and inverse WD (*n* = 408). The data covered a range of climate zones, with the highest number of species affiliated to temperate (*n* = 112–201), followed by wet tropical (*n* = 73–115), subtropical (*n* = 52–83), seasonally dry tropical (*n* = 8–16) and desert or dry shrubland (*n* = 1–8) (Supporting Information Table S1). Similarly, data coverage for the RES traits had the highest number of species in the temperate climate zone (*n* = 198–236), followed by wet tropical (*n* = 95–102), subtropical (*n* = 91–102), seasonally dry tropical (*n* = 12–15) and desert or dry shrubland (*n* = 1–7; Table S1).

**Fig. 2:**
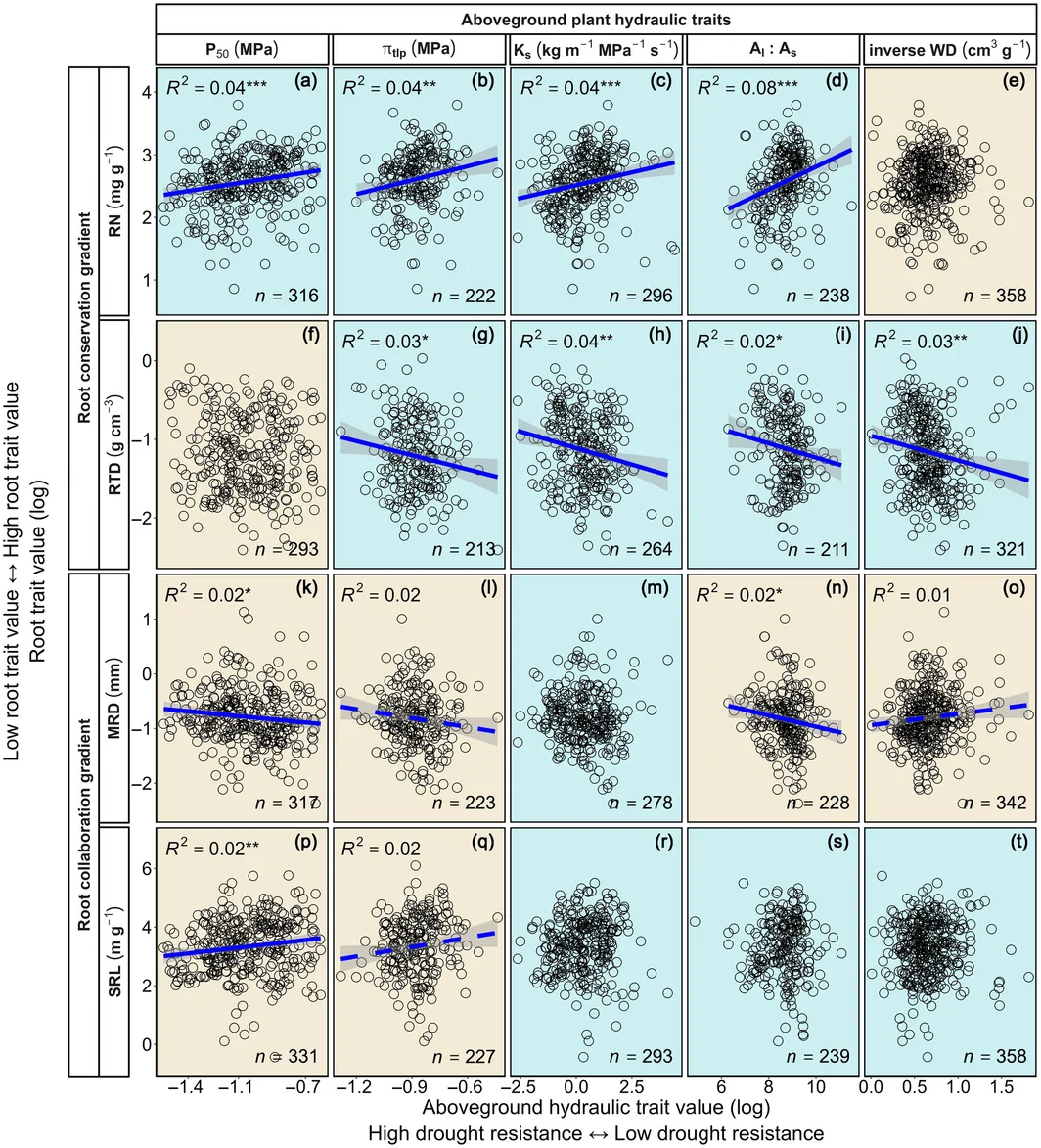
Bivariate relationships (or lack thereof) between the traits of the root economics space and aboveground plant hydraulic traits in woody species. Solid blue lines indicate significant relationships at *P* < 0.05, and dashed blue lines indicate a marginally significant trend (0.05 < *P* < 0.10). Panels without fitted regression lines indicate no significant association. The coefficients of determination (*R* ^2^) of the linear regressions are shown at the top. Significant relationships are denoted by *, *P* < 0.05; **, *P* < 0.01; ***, *P* < 0.001. The shadow around the slope corresponds to the 95% confidence intervals. Number of species (*n*) is given for each panel. The brown background represents a mismatch in either trend or relationship between obtained results and predicted relationships depicted in Fig. 1. The blue background represents a match in either trend or significant relationships between obtained results and predicted relationships depicted in Fig. 1. Note that P_50_ and π_tlp_ values are negative. Columns 1–4 show the relationships of P_50_, π_tlp_, K_s_, A_l_ : A_s_ and inverse WD, respectively, with RN (a–e), RTD (f–j), MRD (k–o) and SRL (p–t). High drought resistance is defined by more negative hydraulic safety traits (P_50_ and π_tlp_) values as well as lower hydraulic efficiency traits (K_s_ and A_l_ : A_s_), whereas low drought resistance is defined by less negative hydraulic safety traits and higher hydraulic efficiency traits values. P_50_, xylem vulnerability to embolism; π_tlp_, leaf water potential at turgor loss point; K_s_, maximum stem xylem‐specific hydraulic conductivity; A_l_ : A_s_, leaf area to sapwood area ratio; inverse WD, inverse wood density; RN, root nitrogen content; RTD, root tissue density; MRD, mean root diameter; and SRL, specific root length.

For woody species, we found most support for the hypothesized relationships between the aboveground hydraulics traits and the fine‐root traits that define the conservation gradient (RN and RTD) of the RES, whereas our expectations regarding the traits associated with the collaboration gradient of the RES were largely unsupported (MRD and SRL; Figs 1, 2, 4). However, all significant relationships explained only a small proportion of trait variance (≤ 8%). Consistent with our expectations, hydraulic safety (P_50_ and π_tlp_) and hydraulic efficiency traits (K_s_ and A_l_ : A_s_) were positively correlated with RN (*R* ^2^ = 0.04 to 0.08, *P* < 0.01 to *P* < 0.001; Fig. 2a–d) and negatively correlated with RTD (*R* ^2^ = 0.02 to 0.04, *P* < 0.05 to *P* < 0.01; Fig. 2g–j). This indicates that species with acquisitive resource strategies in roots – characterized by higher RN and/or lower RTD – are more likely to have acquisitive resource strategies in aboveground hydraulic traits, that is less negative P_50_ and π_tlp_ and higher K_s_ and A_l_ : A_s_. Contrary to our expectations, we found that MRD was negatively correlated with both P_50_ and π_tlp_, indicating that species with thicker roots show a tendency towards higher hydraulic safety (*R* ^2^ = 0.02, *P* < 0.05 to *P* < 0.05 to *P* < 0.001; Fig. 2k,l). Similarly and reflecting the inverse relationship between MRD and SRL, stem P_50_ and leaf π_tlp_ were positively correlated with SRL (*R* ^2^ = 0.02, *P* < 0.01 to *P* < 0.05 to *P* < 0.001; Fig. 2p,q). Additionally, A_l_ : A_s_ was negatively correlated with MRD, showing that species with thicker roots tend to have more sapwood area relative to leaf area (*R* ^2^ = 0.02, *P* < 0.05; Fig. 2n). For nonwoody species, however, the RES traits and the two hydraulic traits (π_tlp_ and stem‐specific density) were not correlated (Fig. S2).

The phylogenetically informed PGLS regression results were consistent with non‐phylogenetic OLS results with comparable explanatory power and trends. In short, PGLS regression also showed that hydraulic safety and hydraulic efficiency traits were positively correlated with RN and negatively correlated with RTD (Fig. S1). In contrast to the OLS regression, PGLS regression showed no relationship between MRD and either P_50_ or inverse WD (Fig. S1k,o).

The results for nonwoody species, with traits limited to π_tlp_ and inverse stem‐specific density (SSD), showed no significant relationships between the RES traits and π_tlp_ or inverse SSD despite sufficient data, particularly for π_tlp_ (*n* = 116). The only exception was that thick‐rooted nonwoody species tended to show lower SSD (*R* ^2^ = 0.09, 0.05 < *P* < 0.10; Fig. S2f).

Further, we examined the relationships between aboveground plant hydraulic traits and the two key traits of the LES: LN content and LDMC (Fig. S3) to test for similarities across functionally equivalent leaf and root traits (LN‐RN and LDMC‐RTD). We found a similar pattern for LN and LDMC to that observed in RN and RTD, respectively. Specifically, P_50_ and π_tlp_ were positively correlated with LN (*R* ^2^ = 0.04 to 0.09, *P* < 0.05 to *P* < 0.001; Fig. S3a,b) while being negatively correlated with LDMC (*R* ^2^ = 0.06 to 0.10, *P* < 0.001; Fig. S3f,g), indicating that species with acquisitive resource strategies in leaves – characterized by higher LN and/or lower LDMC – show a tendency towards lower hydraulic safety. By contrast, K_s_ was positively correlated with LN (*R* ^2^ = 0.29, *P* < 0.001; Fig. S3c) while being negatively correlated with LDMC (*R* ^2^ = 0.02, *P* < 0.05; Fig. S3h), showing that species with acquisitive resource strategies in leaves – characterized by higher LN and/or lower LDMC – show a tendency towards higher hydraulic efficiency. Similar to RN, LN was also positively correlated with A_l_ : A_s_ (*R* ^2^ = 0.10, *P* < 0.001; Fig. S3d) while being negatively correlated with LDMC (*R* ^2^ = 0.04, *P* < 0.05; Fig. S3i), showing that species with higher LN and/or lower LDMC tend to have more leaf area relative to sapwood area. Finally, inverse WD was negatively correlated with LDMC (*R* ^2^ = 0.02, *P* < 0.05; Fig. S3g).

**Fig. 3:**
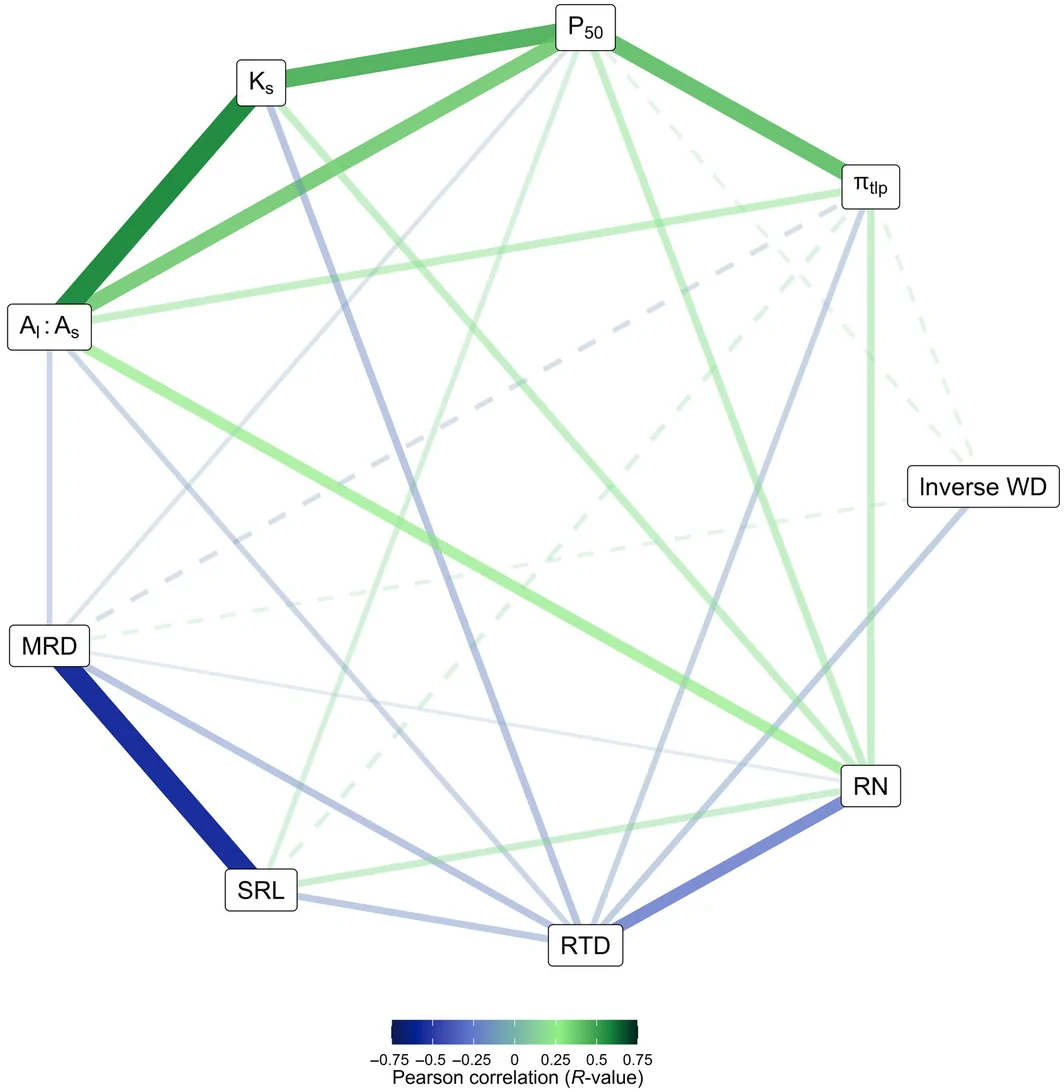
Pairwise correlations of aboveground plant hydraulic traits and the root economics space traits for woody species. Nodes represent the studied traits and line thickness and opacity represent the strength of the correlation. Green and blue lines represent positive and negative correlations, respectively. Solid lines indicate significant Pearson correlations at *P* < 0.05, and dashed lines indicate a marginally significant correlation (0.05 < *P* < 0.10). P_50_, xylem vulnerability to embolism; π_tlp_, leaf water potential at turgor loss point; K_s_, maximum stem xylem‐specific hydraulic conductivity; A_l_ : A_s_, leaf area to sapwood area ratio; inverse WD, inverse wood density; RN, root nitrogen content; RTD, root tissue density; MRD, mean root diameter; and SRL, specific root length. Number of samples per pair was the same as described in Fig. 3. Note that P_50_ and π_tlp_ values are negative.

Unlike the OLS or PGLS regressions, the pairwise correlations enabled us to see the intercorrelation within the hydraulic traits as well as within the RES traits, revealing several significant relationships, both positive (*n* = 15) and negative (*n* = 12) (Fig. 3). In particular, there were strong positive correlations between hydraulic safety traits (P_50_ and π_tlp_; *R* = 0.41, *P* < 0.001) as well as hydraulic efficiency traits (K_s_ and A_l_ : A_s_; *R* = 0.56, *P* < 0.001). We also found strong positive correlations between hydraulic efficiency traits (K_s_ and A_l_ : A_s_) and P_50_ as a hydraulic safety trait (*R* = 0.45–0.38; *P* < 0.001), indicating a strong trade‐off between hydraulic efficiency (represented by high K_s_ and A_l_ : A_s_) and safety (represented by more negative P_50_; Fig. 3). However, there was a weaker correlation between the other hydraulic safety trait, π_tlp_ and A_l_ : A_s_ (*R* = 0.20, *P* < 0.001). In addition, there were weak positive correlations between the inverse WD and both P_50_ and π_tlp_ as hydraulic safety traits (*R* = 0.11, 0.05 < *P* < 0.10, respectively). Inverse WD was independent of hydraulic efficiency traits (K_s_ and A_l_ : A_s_). Also, K_s_ and π_tlp_ were independent of each other (Fig. 3). In line with the RES, there were negative correlations between traits of the collaboration gradient (MRD and SRL; *R* = −0.55, *P* < 0.001) as well as between traits of the conservation gradient (RN and RTD; *R* = −0.31, *P* < 0.001), although the correlation between the collaboration gradient traits was much stronger compared to the conservation gradient traits (Fig. 3). In addition, RTD was negatively correlated with both SRL and MRD (*R* = −0.18 to −0.19, *P* < 0.001), whereas RN and SRL were positively correlated (*R* = 0.18, *P* < 0.001). Supporting the bivariate relationships (Fig. 2), hydraulic safety (P_50_ and π_tlp_) and efficiency traits (K_s_ and A_l_ : A_s_) were positively correlated with RN (*R* = 0.19 to 0.28, *P* < 0.001). By contrast, π_tlp_, K_s_, A_l_ : A_s_, and inverse WD were negatively correlated with RTD (*R* = −0.15 to −0.19, *P* < 0.05 to *P* < 0.01), aligning with bivariate regression (Fig. 2). Additionally, P_50_, π_tlp_ and A_l_ : A_s_ were weakly negatively correlated with MRD (*R* = −0.12 to −0.15, 0.05 < *P* < 0.10 to *P* < 0.05), while inverse WD was marginally positively correlated with MRD (*R* = 0.11, 0.05 < *P* < 0.10). Finally, similar to the bivariate relationships (Fig. 2), only hydraulic safety traits (P_50_ and π_tlp_) showed weak positive correlation with SRL (Fig. 3; *R* = 0.13 to 0.15, *P* < 0.01 to 0.05 < *P* < 0.10, respectively).

## Discussion

**Fig. 4:**
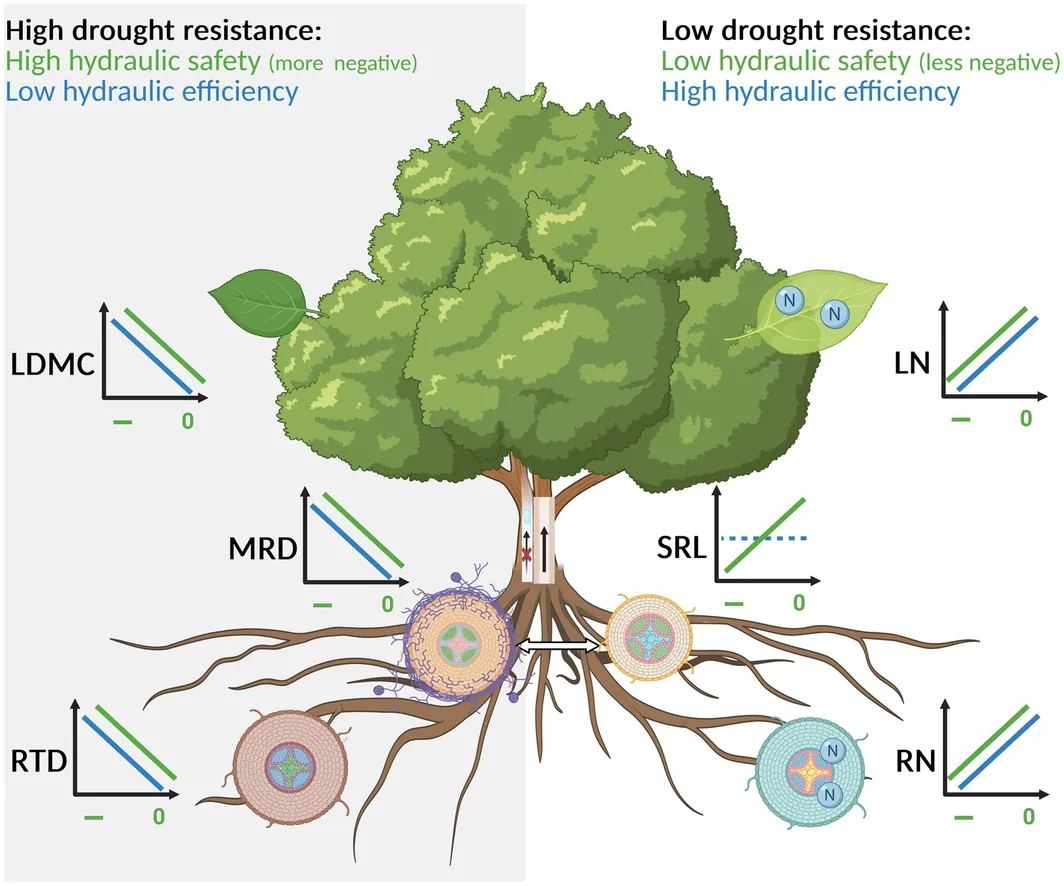
Conceptual summary figure shows the results of the relationships between aboveground plant hydraulic strategies and root economics space (RES) traits. The figure is divided in (i) low drought resistance strategy on the right with low hydraulic safety (P_50_ and π_tlp_) and high hydraulic efficiency (K_s_ and A_l_ : A_s_) and (ii) high drought resistance on the left with high hydraulic safety and low hydraulic efficiency. Relationships between plant hydraulic traits and the RES traits are shown as bivariate relationships where green/blue lines depict the relationships between hydraulic safety/hydraulic efficiency and RES traits, respectively. Dashed lines indicate a nonsignificant trend. LN, leaf nitrogen content; LDMC, leaf dry matter content; MRD, mean root diameter; RN, root nitrogen content; RTD, root tissue density; SRL, specific root length. This figure was created in BioRender, Weigelt, A. (2026) (BioRender.com/g1pfibb).

Considering a suite of hydraulic traits related to water uptake, use and transport, we aimed to determine the relationships between aboveground hydraulic adaptations to drought stress and fine‐root traits of the RES. This is important because roots are the primary organ to take up and transport water and have not been directly linked in an integrative way to aboveground water use and transport. We found distinct relationships between aboveground hydraulic traits and fine‐root traits reflecting the conservation and collaboration gradients. In particular, woody species with more acquisitive fine‐root traits (higher RN) tend to have lower hydraulic safety (less negative P_50_ and π_tlp_) but higher hydraulic efficiency (K_s_ and A_l_ : A_s_) traits (Figs 2, 3, 4). Conversely, woody species with conservative fine‐root traits – higher RTD – tend to have higher drought resistance as indicated by the higher (more negative) hydraulic safety and lower hydraulic efficiency traits (Figs 2, 3, 4). However, we did not find support for our hypothesis that plant species with larger diameter roots would be less drought‐resistant (higher efficiency but low safety traits) due to a higher risk of absolute root shrinkage (or other mechanisms). Rather, we found that woody species with thicker roots tended to be more drought‐resistant than those with thinner roots, possibly highlighting the importance of mycorrhizal association improving water and nutrient absorption in thick‐rooted species (Bergmann *et al*., 2020). However, the overall relationships between aboveground hydraulic traits and root traits suggest that the RES traits are either only weakly involved in plant drought response strategies, or that their role in drought response is at least partly independent from aboveground adaptations. This might explain variability in plant strategies to cope with drought, where aboveground and belowground adaptations may be decoupled.

Consistent with our expectation, we found that higher RN, which corresponds to the acquisitive end of the conservation gradient, was correlated with lower hydraulic safety (less negative P_50_ and π_tlp_) and higher hydraulic efficiency (higher K_s_ and A_l_ : A_s_) traits (Fig. 2). According to resource economics theory, RN is thought to be associated with high metabolic activity and low construction costs (Reich, 2014; Bergmann *et al*., 2020), resulting in a higher growth rate but a shorter lifespan. Thus, plant species with higher RN that have a fast return on carbon investment are associated with higher hydraulic conductivity and less drought‐resistant stems and leaves. In particular, less negative hydraulic safety and higher hydraulic efficiency traits were associated with less carbon‐costly roots, suggesting woody species with lower structural investments in roots are associated with higher embolism formation in xylem as found for aboveground organs in poplars (Harvey & van den Driessche, 1997). More specifically, plant species with higher hydraulic conductivity and leaf area to sapwood area ratio have a higher water demand, which likely increases their embolism formation risk during drought stress (Mencuccini *et al*., 2019). In line with this, we observed a similar pattern for LN – which is often related to RN (Reich, 2014; Weigelt *et al*., 2021) – but with a stronger magnitude (Fig. S3). Our results corroborate previous studies showing a negative relationship between LN and P_50_ and positive relationships between LN and both K_s_ and A_l_ : A_s_ (D. Liu *et al*., 2024). Overall, our findings, despite their small explanatory power, indicated that woody species with higher RN are likely to be less efficient at conserving water under drought conditions. Woody species with higher efficiency in water transport (higher K_s_ and A_l_ : A_s_) are associated with roots possessing higher RN, which can be built at low carbon cost. This supports previous findings of a balance between water loss and carbon fixation (Tyree & Zimmermann, 2013).

Similarly, we found that for woody species a conservative fine‐root strategy – higher RTD – was negatively correlated with π_tlp_ (more negative), K_s_, A_l_ : A_s_, and inverse WD (Fig. 2); this was also evidenced by the overall network correlation (Fig. 3). From an aboveground perspective, plant species with lower K_s_ and A_l_ : A_s_ but a higher WD invest a higher amount of carbon in their structural organs, resulting in lower transport efficiency and thus higher drought resistance (Mencuccini *et al*., 2019). Moreover, plant species with higher RTD tend to occur and survive in lower water and soil nutrient availability conditions (Kramer‐Walter *et al*., 2016; Laughlin *et al*., 2021). Thus, lower K_s_, A_l_ : A_s_ and WD, whereas more negative π_tlp_ in woody species with higher RTD, again underscore that higher tissue density in roots is weakly associated with a lower water transport efficiency but higher safety (more negative π_tlp_) and thus higher drought resistance (Hacke *et al*., 2000). To complement these results, we found a positive association of LDMC, as a conservative leaf trait, with hydraulic safety traits but negative associations with hydraulic efficiency traits, corroborating a previous study (D. Liu *et al*., 2024). Taken together, our results indicate that water conservation aboveground is related to high carbon construction costs aboveground and belowground (LDMC and RTD). As such, woody species with higher RTD are more efficient at conserving water status in drought conditions, owing to their lower hydraulic conductivity and higher (more negative) hydraulic safety.

In partial contrast with our expectations, but consistent with weak trade‐offs between hydraulic safety and efficiency in woody species (Gleason *et al*., 2016), we documented a weak tendency of thick‐rooted woody species to have higher hydraulic safety (more negative P_50_ and π_tlp_) and lower hydraulic efficiency (A_l_ : A_s_; Figs 2, 3). Our hypotheses emphasized the potential of (1) thicker cortex tissues (and thus thicker roots) to be associated with greater root shrinkage (and thus lower need for and investment in aboveground hydraulic safety) and (2) larger diameter conduits (and thus thicker roots) to have higher hydraulic conductance (i.e. efficiency). However, root shrinkage could also be proportional to root surface area to volume ratios (Boldrin *et al*., 2018), which are lower for thicker roots; this may explain why both thin‐ and thick‐rooted species can have diverse values of hydraulic safety, with thick‐rooted woody species being slightly more likely to be paired with more negative stem P_50_ and π_tlp_. Further, although thick‐rooted species are more likely to have higher rates of axial water transport through the stele due to larger conduits (O'Keefe *et al*., 2022), they are also likely to have lower rates of radial water transport (from soil into the stele) due to greater stele thickness (Steudle & Peterson, 1998; Huang & Eissenstat, 2000). Thus, one aspect of root anatomical features of thick roots favours hydraulic efficiency while another aspect hinders it. This duality may explain why both thin‐ and thick‐rooted species have diverse values of K_s_ and A_l_ : A_s_, but with thick‐rooted species being somewhat more likely to have low A_l_ : A_s_. The higher hydraulic safety traits in thick‐rooted woody species could also be attributed to the higher proportional cortical space in these species, which generally provides more space for mycorrhizal colonization (Comas *et al*., 2012; Sanaei *et al*., 2025). Specifically, the branched hyphae of mycorrhizal fungi facilitate nutrient and water uptake through a larger surface area and larger soil volume (Moora *et al*., 2025). As SRL is opposite to MRD (*R* = −0.55; Fig. 3), the counteracting roles of root anatomical traits discussed above may also explain the weak positive correlation we observed between SRL and aboveground hydraulic safety traits (P_50_ and π_tlp_; Figs 2, 3).

In contrast to woody species, nonwoody species did not show significant relationships between the two available aboveground plant hydraulic traits – π_tlp_ and inverse SSD – and the RES traits (Fig. S2). There might be two potential explanations for this result. First, while the basic mechanisms of water absorption and transportation in woody and nonwoody species are similar, these plant types differ substantially in height, size and longevity, as well as in aboveground and belowground phenology, morphology and anatomy. This might lead to varied plant strategies towards drought resistance between woody and nonwoody species (Volaire, 2018; Májeková *et al*., 2021; R. Huang *et al*., 2024). Second, while our nonwoody species data includes species with different life spans (annual and perennial) and functional groups (grass, forb and legume), most species are temperate (*n* = 103), with only a few species from desert or dry shrubland (*n* = 6) and subtropical (*n* = 2) climate zones; this might reduce trait variation and result in a reduced probability to find a significant relationship.

### Conclusion

Although the relationships we observed between hydraulic traits and root traits that define the conservation gradient of the RES were often in the direction we hypothesized, all of the relationships explained only a low amount of variance (≤ 8%; Figs 2, 3). This weak nature of coordination between RES traits and aboveground hydraulic traits shows that species with very similar root economics traits can have vastly differing aboveground strategies to cope with drought and that adaptations to drought stress might be largely decoupled aboveground and belowground. Such flexibility in pairing aboveground hydraulic phenotypes with belowground economic phenotypes may be a key factor that allows niche differentiation and functional diversification among species, thus promoting biodiversity at community and ecosystem scales (Laughlin, 2014). The weak links between economic traits of fine roots and aboveground hydraulic traits might not be surprising given: (1) the complex, multidimensional nature of plant strategies for acquiring and using water, and for resisting negative effects of water scarcity (Comas *et al*., 2013; Weigelt *et al*., 2021; Torres‐Ruiz *et al*., 2024); (2) that none of the RES traits have been conceptually or empirically considered as primary, or direct, adaptations related to water stress or hydraulic conductance; and (3) the effect of fine root traits on plant performance (e.g. resource uptake, drought resistance) is further interacting with other plant traits not covered by RES (e.g. root to shoot biomass ratios, root to leaf surface area ratios, rooting depth or depth of water acquisition). Further, because acquisition and use of carbon and nutrients, which are the foci of individual root economic traits and of the axes of the RES (Bergmann *et al*., 2020), *requires* water acquisition and use, our expectation persists for *indirect*, even if weak, links between root economic space traits and aboveground plant hydraulic traits. Thus, while our study provides first insights on understanding whole‐plant strategies in response to drought, we acknowledge the need for further research integrating root physiology, anatomy, architecture and hydraulic traits to better understand whole‐plant water relations.

## Competing interests

None declared.

## Author contributions

Conceptualization and workshop participation: AS, SS, KEM, MLM, BS, DCL, BHPR, GTF, SDA‐D, KEB, JB, NE, FCJ, HP, HOV, JDV, MW, LM and AW. AS performed data compilation and statistical analysis. AS, SS and AW led the writing of the manuscript. All the co‐authors commented on analytical approaches and contributed to editing the draft and final manuscripts multiple times. AS and SS contributed equally to this work.

## Disclaimer

The New Phytologist Foundation remains neutral with regard to jurisdictional claims in maps and in any institutional affiliations.

## Supporting information


**Dataset S1** Species‐level mean values of aboveground plant hydraulic, fine‐root, and leaf traits for woody and non‐woody species used in this study.

**Fig. S1** Phylogenetic generalized least squares relationships between the traits of the root economics space and plant hydraulic traits in woody species.
**Fig. S2** Bivariate relationships between the root economics space and hydraulic traits for nonwoody species.
**Fig. S3** Bivariate relationships between plant hydraulic traits and the main two leaf economics spectrum traits of woody species.
**Table S1** Summary of aboveground plant hydraulic and fine‐root traits across climate zones.
Please note: Wiley is not responsible for the content or functionality of any Supporting Information supplied by the authors. Any queries (other than missing material) should be directed to the *New Phytologist* Central Office.

## Data Availability

The data supporting this study are available in Dataset S1.
