# Supplementary material for: On the relationship between root economics and plant hydraulic traits
Source: New Phytol. 2026 Jul 13;251(6):3289–302. doi: 10.1111/nph.71442 (PMC13491273; doi:10.1111/nph.71442)
Supplement: Supplementary file 1 [file NPH-251-3289-s002.pdf]

New Phytologist

New Phytologist Supporting Information

Article title: On the relationship between root economics and plant hydraulic traits

Authors: Anvar Sanaei, Srijna Saxena, Kevin E. Mueller, M. Luke McCormack, Bernhard Schuldt, Daniel C. Laughlin, Bruno H.P. Rosado, Gregoire T. Freschet, Shalom D. Addo-Danso, Kathryn E. Barry, Joana Bergmann, Nico Eisenhauer, Florentin Clemens Jaeger, Hendrik Poorter, Harry Olde Venterink, Jorad De Vries, Monique Weemstra, Liesje Mommer, Alexandra Weigelt

Article acceptance date: 26 June 2026

The following Supporting Information is available for this article:

Fig. S1. Phylogenetic generalized least squares relationships between the traits of the root economics space (RES) and plant hydraulic traits in woody species.
Fig. S2. Bivariate relationships between the RES and hydraulic traits for nonwoody species.
Fig. S3. Bivariate relationships between plant hydraulic traits and the main two leaf economics spectrum traits of woody species.
Table S1. Summary of above-ground plant hydraulic and fine-root traits across climate zones.

Fig. S1 Phylogenetic generalized least squares (PGLS) relationships (or lack thereof) between the traits of the root economics space and aboveground plant hydraulic traits in woody species. Solid lines indicate significant relationships at $P < 0.05$, and dashed lines indicate a marginally significant trend ($0.05 < P < 0.10$). Panels without fitted regression lines indicate no associations. The coefficients of determination (R^2) of the linear regressions are shown at the top. Significant relationships are denoted by * ($P < 0.05$), ** ($P < 0.01$) and *** ($P < 0.001$). Number of species (n) is given for each panel. The brown background represents a mismatch in either trend or significant relationships between obtained results and predicted relationships depicted in Fig. 1. The blue background represents a match in either trend or significant relationships between obtained results and predicted relationships depicted in Fig. 1. Abbreviations: P_{50}, xylem vulnerability to embolism; π_{tlp}, leaf water potential at turgor loss point; K_s, maximum stem xylem-specific hydraulic conductivity; A_l:A_s, leaf area to sapwood area ratio; inverse WD, inverse wood density; RN, root nitrogen content; RTD, root tissue density; MRD, mean root diameter; and SRL, specific root length. Note that P_{50} and π_{tlp} values are negative. High drought resistance is defined by more negative hydraulic safety traits (P_{50} and π_{tlp}) values as well as lower hydraulic efficiency traits (K_s and A_l:A_s), whereas low drought resistance is defined by less negative hydraulic safety traits and higher hydraulic efficiency traits values. Columns 1–4 show the relationships of P_{50}, π_{tlp}, K_s, A_l:A_s, and inverse WD, respectively, with RN (a, b, c, d, e), RTD (f, g, h, i, j), MRD (k, l, m, n, o), and SRL (p, q, r, s, t).

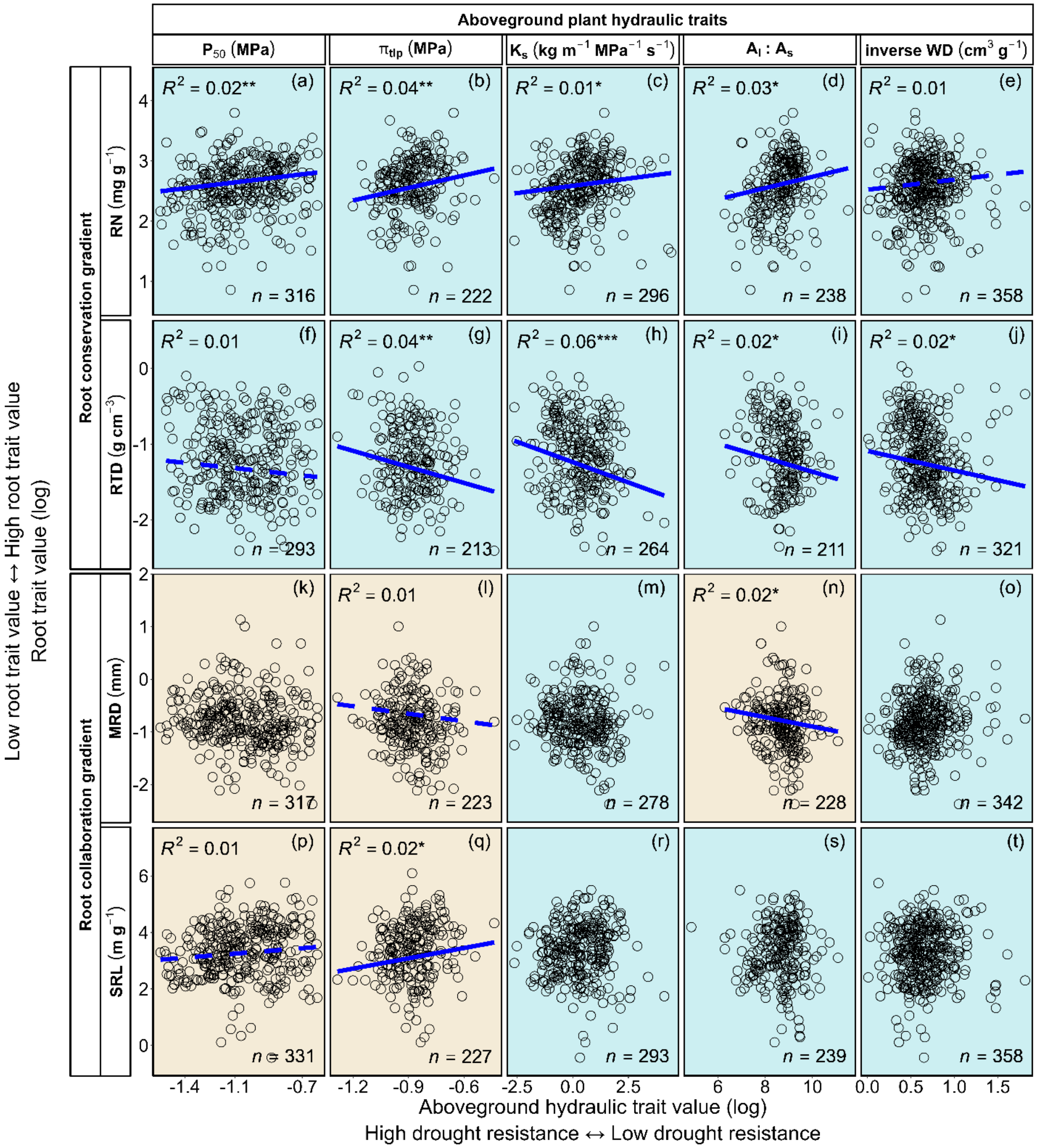

Aboveground plant hydraulic traits
P50 (MPa)
πtlp (MPa)
Ks (kg m−1 MPa−1 s−1)
Al : As
inverse WD (cm3 g−1)
Root conservation gradient
Root collaboration gradient
RN (mg g−1)
RTD (g cm−3)
MRD (mm)
SRL (m g−1)
Low root trait value ↔ High root trait value
Root trait value (log)
(a) R2 = 0.02** n = 316
(b) R2 = 0.04** n = 222
(c) R2 = 0.01* n = 296
(d) R2 = 0.03* n = 238
(e) R2 = 0.01 n = 358
(f) R2 = 0.01 n = 293
(g) R2 = 0.04** n = 213
(h) R2 = 0.06*** n = 264
(i) R2 = 0.02* n = 211
(j) R2 = 0.02* n = 321
(k) n = 317
(l) R2 = 0.01 n = 223
(m) n = 278
(n) R2 = 0.02* n = 228
(o) n = 342
(p) R2 = 0.01 n = 331
(q) R2 = 0.02* n = 227
(r) n = 293
(s) n = 239
(t) n = 358
Aboveground hydraulic trait value (log)
High drought resistance ↔ Low drought resistance

New Phytologist

Fig. S2 Bivariate relationships between the root economics space and hydraulic traits for nonwoody species. Solid lines indicate significant relationships at $P < 0.05$, and dashed lines indicate a marginally significant trend ($0.05 < P < 0.10$). Panels without fitted regression lines indicate no associations. The coefficients of determination (R^2) of the linear regressions are shown at the top. Significant relationships are denoted by * ($P < 0.05$), ** ($P < 0.01$) and *** ($P < 0.001$). The shadow around the slope corresponds to the 95% confidence intervals. Number of species (n) is given for each panel. Abbreviations: π_{tlp}, leaf water potential at turgor loss point; inverse SSD, inverse stem-specific density; RN, root nitrogen content; RTD, root tissue density; MRD, mean root diameter; and SRL, specific root length. Note that π_{tlp} values are negative. Columns 1–2 show the relationships of π_{tlp} and inverse SSD, respectively, with RN (a, b), RTD (c, d), MRD (e, f), and SRL (g, h).

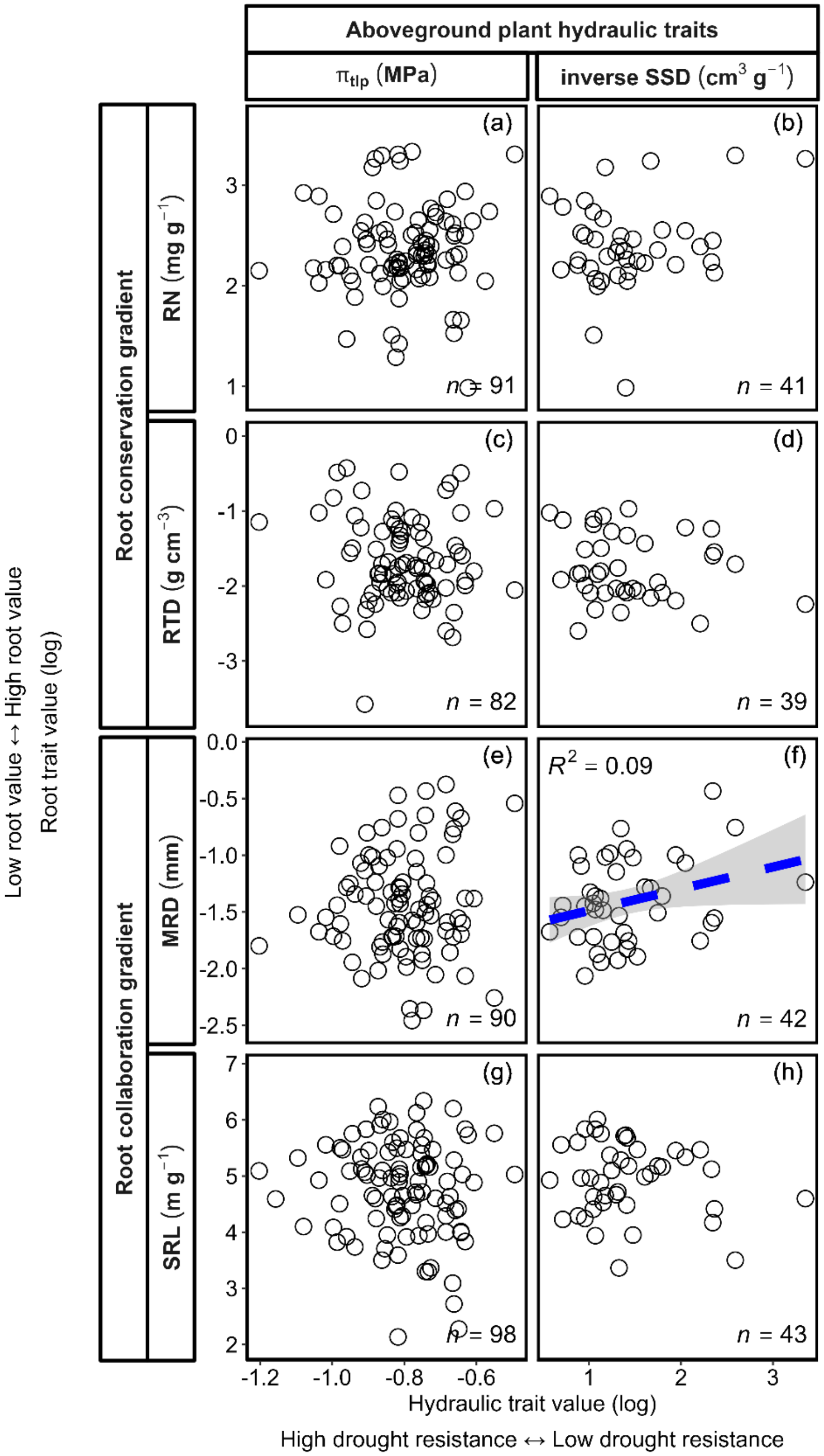

Aboveground plant hydraulic traits
π_{tlp} (MPa)
inverse SSD (cm^3 g^{-1})
Root conservation gradient
Root collaboration gradient
RN (mg g^{-1})
RTD (g cm^{-3})
MRD (mm)
SRL (m g^{-1})
(a) (b) (c) (d) (e) (f) (g) (h)
n = 91
n = 41
n = 82
n = 39
n = 90
n = 42
n = 98
n = 43
R^2 = 0.09
Low root value ↔ High root value
Root trait value (log)
Hydraulic trait value (log)
High drought resistance ↔ Low drought resistance

Fig. S3

Fig. S3 Bivariate relationships between plant hydraulic traits and the main two leaf economics spectrum traits of woody species. Solid lines indicate significant relationships at $P < 0.05$, and dashed lines indicate a marginally significant trend ($0.05 < P < 0.10$). Panels without fitted regression lines indicate no associations. The coefficients of determination (R^2) of the linear regressions are shown at the top. Significant relationships are denoted by * ($P < 0.05$), ** ($P < 0.01$) and *** ($P < 0.001$). The shadow around the slope corresponds to the 95% confidence intervals. Number of species (n) is given for each panel. Abbreviations: P_{50}, xylem vulnerability to embolism; π_{tlp}, leaf water potential at turgor loss point; K_s, stem xylem hydraulic conductivity; A_l:A_s, leaf area to sapwood area ratios; inverse WD, inverse wood density; LN, leaf nitrogen content; and LDMC, leaf dry matter content. Note that P_{50} and π_{tlp} values are negative. Columns 1–4 show the relationships of P_{50}, π_{tlp}, K_s, A_l:As, and inverse WD, respectively, with LN (a:e), and LDMC (f:j).

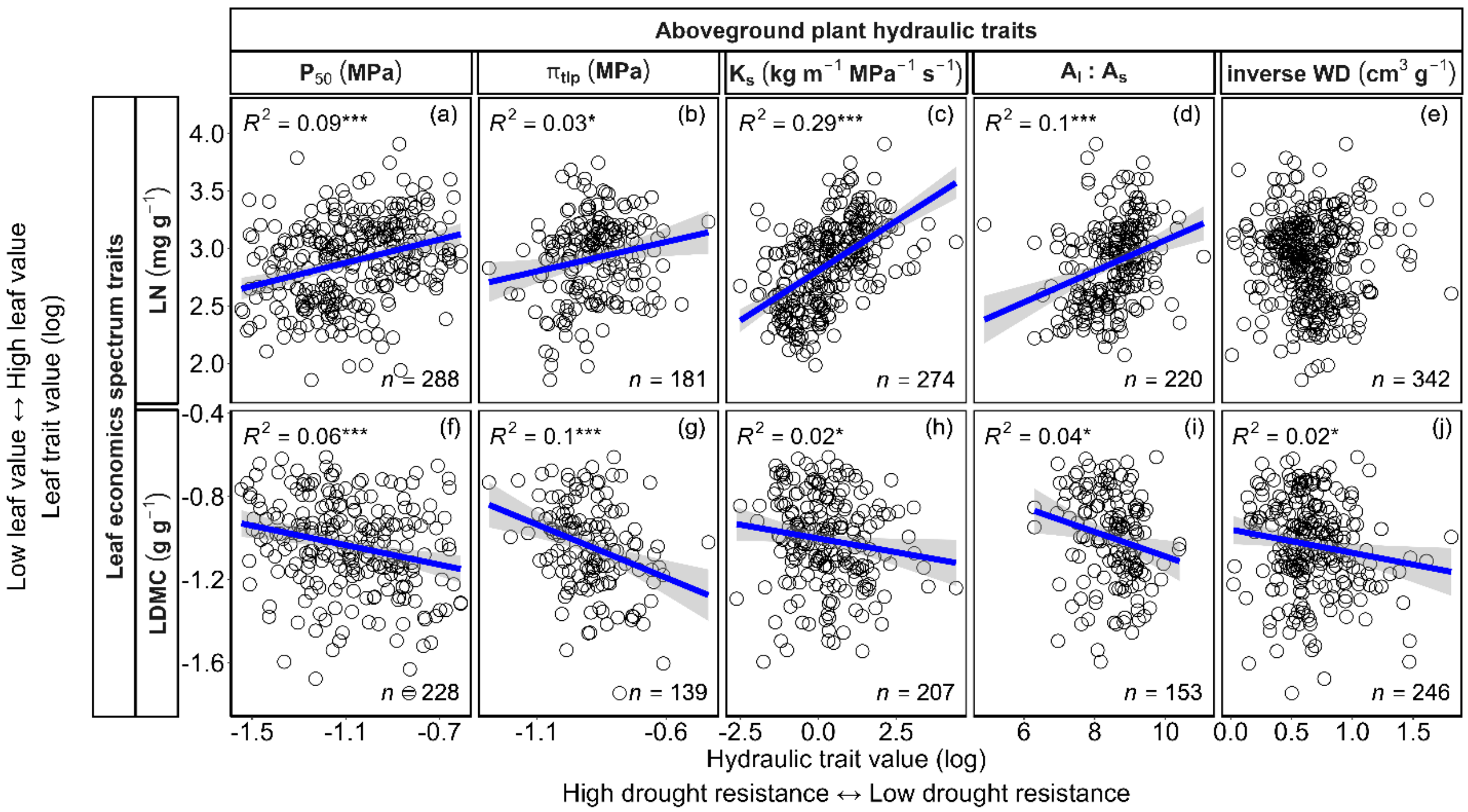

New Phytologist

Table S1 Summary of aboveground plant hydraulic and four core root traits within the root economics space for woody species across climate zones. Mean ± standard error is shown, and number of species per climate zone is shown within the parenthesis. Abbreviations: P_{50}, xylem vulnerability to embolism; π_{tlp}, leaf water potential at turgor loss point; K_s, stem xylem hydraulic conductivity; A_l:A_s, leaf area to sapwood area ratios; inverse WD, inverse wood density; RN, root nitrogen content; RTD, root tissue density; MRD, mean root diameter; and SRL, specific root length.

Aboveground plant hydraulic traits					
Climate zone	**P_{50} (MPa)**	**π_{tlp} (MPa)**	**K_s (kg m^{-1} MPa^{-1} s^{-1})**	**A_l:A_s**	**inverse WD (cm^3 g^{-1})**
temperate	−3.39±0.12 (190)	−1.85±0.05 (112)	1.81±0.14 (152)	6263.65±466.00 (120)	0.52±0.01 (201)
subtropical	−3.73±0.21 (83)	−2.01±0.08 (52)	1.12±0.13 (65)	5937.34±677.39 (54)	0.54±0.02 (71)
wet tropical	−2.55±0.14 (74)	−1.70±0.04 (73)	6.67±1.51 (86)	11028.89±1486.56 (80)	0.54±0.01 (115)
seasonally dry tropical	−2.08±0.36 (8)	−2.11±0.13 (10)	5.43±1.04 (14)	11998.66±2306.40 (11)	0.54±0.04 (16)
desert or dry shrubland	−4.49±1.12 (8)	−1.67 (1)	0.75±0.16 (8)	1498.24±512.67 (7)	0.81±0.09 (5)
Total number species	363	248	325	272	408

Fine-root traits				
Climate zone	**RN (%)**	**RTD (g cm^{-3})**	**MRD (mm)**	**SRL (m g^{-1})**
temperate	13.93±0.38 (236)	0.33±0.01 (198)	0.51±0.02 (213)	49.02±3.47 (233)
subtropical	13.86±0.59 (91)	0.38±0.02 (97)	0.49±0.02 (102)	36.21±3.25 (100)
wet tropical	14.83±0.73 (95)	0.36±0.01 (97)	0.47±0.03 (101)	37.83±3.48 (102)
seasonally dry tropical	15.30±1.62 (15)	0.40±0.06 (12)	0.59±0.10 (14)	43.67±13.45 (14)
desert or dry shrubland	18.60±7.23 (4)	0.46 (1)	0.43±0.05 (2)	92.40±32.85 (7)
Total number species	441	405	432	456
